# A Narrative Review of Wild and Semiwild Edible Plants in Ethiopia: Agroecological Perspectives, Ethnic Diversity, Proximate Composition, and Phytochemical Analysis

**DOI:** 10.1155/ijfo/2265433

**Published:** 2025-07-22

**Authors:** Derebe Alemneh

**Affiliations:** Department of Biology, College of Natural and Computational Science, Injibara University, Bahir Dar, Amhara, Ethiopia

**Keywords:** antioxidants, minerals, neutraceuticals, proximate composition, wild edible plants

## Abstract

All over the world, wild edible plants are predominantly the sources of famine food. Ethiopia, with food-insecure populations, needs such food-supplementing plant groups. The current review article is aimed at presenting a compiled list of the species, showing the diversity of the species, and elaborating on their major threats and values. Scientific articles with an ethnobotanical approach that were published from 2004 to 2025 in Ethiopia were gathered. An inclusive review was performed by using a reputable database, Google Scholar. Several phases of screening were conducted. A total of 59 published articles were reviewed. From the published documents, important information was extracted. The gathered data was entered into Microsoft Excel 2010 and analyzed. A total of 665 species (114 families) were recorded. *Cordia africana*, *Syzygium guineense*, and *Ximenia americana* were the three highly mentioned species. Fabaceae was an outlayer family with 63 species. Rubiaceae (31 species) was the second, followed by Malvaceae (28 species). Trees were the dominant species, followed by shrubs, and the preferred edible part was the fruit. There was a record difference across one region to the other, and the southern region was the first in species record (113 species). They were the sources of proteins, lipids, carbohydrates, fibers, vitamin C, and minerals. They were further recorded as nutraceuticals, antioxidants, and income-generating plants. Two major problems were observed: lack of utilising the species as a staple food and low marketability. Nowadays, the species are under several threats. Therefore, significant conservation strategies should be conducted and implemented throughout the country along with promoting their value to the local community.

## 1. Introduction

Food insecurity is the main global concern that results in the search for an alternative food source [1]. Wild edible plants are one of the major substitute food sources for the increasing world's population [2]. By definition, such major alternatives are noncultivated and nondomesticated food sources [3–6]. Apart from the increased population, the world is continually affected by natural disasters such as crop diseases and civil war, and other conflicts have resulted in famine [7]. Some potato-dependent countries of Europe, such as Ireland, Poland, and Portugal, are among several countries of the world that were affected by such phenomena. During that time, wild edible plants were mentioned as the major sources of food for the food-insecure population [7]. It might be due to one or more reasons; such edibles are cited to be consumed throughout the world, in Asian countries [8], such as China [9], India [10], and the Himalayas [11]. In India, about 800 species of wild edibles were recorded to be used as a source of food [12]. South Americans [13], North Americans [14], and African countries such as Kenya [15–18], Cameroon [19], South Africa [20], Sudan [16], and Ethiopia [16, 21–27] used wild edibles as sources of food during food insecurity. Literature further showed that the global utilization of the edibles is predominantly used as famine food sources, and their utilization as a staple food is quite poor [28, 29].

Wild edible plants are not only used to relieve mere hunger but also are a rich source of nutrition, such as proteins, carbohydrates, lipids [24, 30–32], minerals [30, 33], and vitamins [30, 34]. They are rich sources of antioxidants such as phenols, flavonoids, and fibers [30]. Some literature sources showed that wild edibles are healthier than the processed food that can negatively affect human health and result in malnutrition that becomes a global threat [35]. In addition, wild edible plants are a healthy alternative to cultivated vegetables because the cultivated ones might be rich in pesticides and other chemicals [35, 36]. Thus, the global consumption of wild food sources as mere famine food should be corrected, and wild food should be presented as staple and supplementary food in food tables.

As part of the world, Ethiopia is one of the several countries that are repeatedly affected by drought, which results in famine. Civil war, population growth, and poor agricultural practices with low productivity are the other major factors that led the country to be food insecure. For the reason that the population of the country in its several parts is dependent on wild edibles as emergency, supplementary, seasonal, and famine food sources to prevent food insecurity in their households [30], indicating that wild food sources have the ability to supplement common crops at the national level during crises. Studies further showed that wild edibles have an ability to stabilize the country's household resilience [37].

Nowadays, plentiful ethnobotanical works are underway in search of wild edible plants' role, especially for rural communities of Ethiopia. Likewise, most recently, phytochemical and proximate composition studies are being conducted in parallel in several institutions, including universities. Because of such works, the great role of wild edible plants, which was limited traditionally, is now becoming evident to the scientific community. Besides, such works are becoming a motivating action for further works to be conducted regarding the wild edibles. However, there are no sufficient review articles compiling such works showing the compiled abundant wild edible plant resources of the country. As an additional point, knowledge regarding utilizing wild edible plants and their nutritional values should be introduced to the Ethiopian community. The current review paper tried to present the current total record of wild edible plant species of the country and the great role of the species in income generation and as sources of nutrients, phytochemicals, minerals, and medicine, along with the different participating ethnic communities, by reviewing a number of literature sources.

## 2. Objectives of the Review Article

The review article had three principal objectives: (1) Presenting a compiled list of wild edible plants by reviewing previously conducted research works in different parts of Ethiopia for the purpose of showing the wild edible plant wealth of the country for future food security and promoting conservation efforts; (2) showing the diversity of the country's wild edible plants in terms of species, growth form, edible part, region, and agroclimatic zone and explaining the major stakeholder communities of wild edible plants in the country; and (3) elaborating on the major threats to wild edible plants and their value in income generation as a source of medicine, food, antioxidants, and phytochemicals.

## 3. Methodology

### 3.1. Data Gathering

Scientific articles with an ethnobotanical approach that were published from 2004 to 2025 and presented a list of wild edible plants occurring in Ethiopia were gathered. All the gathered articles were original research articles. An inclusive review was performed by using a reputable database, Google Scholar. The search keywords (search words) utilized for the review from Google Scholar were “Wild edible plants in Ethiopia”, “Wild edible plants in southern Ethiopia,” and “Wild and semi-wild edible plants in southern Ethiopia”. Then, only articles conducted on wild edible plants within Ethiopia that are appropriate for the title and that were fit to be used as data sources for the current review paper were considered to be appropriate for inclusion.

### 3.2. Screening and Exclusion Criteria

Several phases of screening were conducted: (1) Duplicates, that is, articles found more than once, were excluded. (2) The abstract of each article was read, and articles without an ethnobotanical approach were excluded. (3) Review articles and studies that were not published, and short communications were excluded. (4) Articles that did not present a list of species and those that did not present the scientific names of the species were excluded. (5) In cases of two or more studies conducted in the same community, only the one that contained more complete information was included.

### 3.3. Data Extraction and Analysis

For the achievement of the objectives of the review, 59 published articles were reviewed. Following the process, 50 published articles and one proceeding were used as a data source. An additional eight published articles were used as a data source for the purpose of analyzing the various roles of wild edible plants. From the published documents, important information was extracted. The check-up was done for the purpose of avoiding repetition of information. The scientific name, family name, local name, edible part, and site of the study were among the important information extracted. The scientific name of each species was cross-checked by using the Flora Books of Ethiopia and Eritrea (Flora of Ethiopia and Eritrea, volumes 1–8). The gathered data was entered into Microsoft Excel 2010 and analysed, and the results were presented in the form of tables, plots, and graphs.

## 4. Findings of the Review

### 4.1. Species Diversity

**Figure 1:**
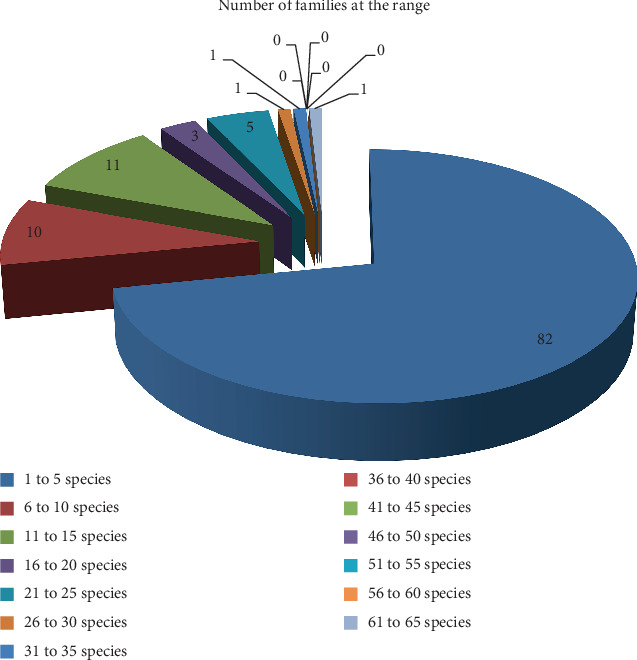
Comparison of family categories in species number.

**Figure 2:**
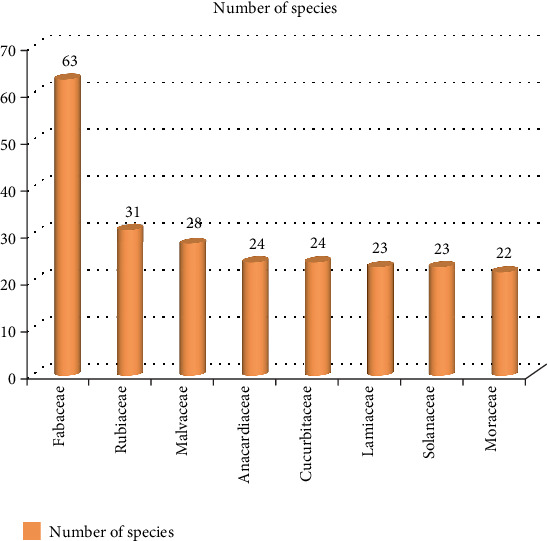
Comparison of top eight families with the highest species.

A total of 665 species of wild edible plants, which were grouped under 114 families, were recorded from the reviewed papers (Table 1). It is a little bit more than the previous reviewed records of [38] (413 species records) and [39] (651 records), respectively. The present record is far from the latter by 14 species. Ethiopia is known to have the highest record of higher plant species, which is estimated to be 6027 vascular plant species [40]. The current wild edible plant record accounts for almost 11% of the country's vascular plant diversity, which shows the country's wild edible plant wealth beyond the total flora diversity. Taxonomically, eight families possess more diverse species of wild edible plants. Fabaceae is the first family in species number (63 species, 9.5%), followed by Rubiaceae (31 species, 4.4%), Malvaceae (28 species, 4.2%), Anacardiaceae and Cucurbitaceae (24 species each), Lamiaceae and Solanaceae (23 species each), and Moraceae (22 species) (Table 1 and Figures 1 and 2). Fabaceae is not only the leading family but also an outlier family with a species range of 36–65 species. No family is found at this range (Figure 1). Most of the families (80 families, 72%), out of the total 114 families, consist of species at the range of 1–5 species (Figure 1). This showed that most of the wild food was gathered from the species that were grouped in a few number of wild edible plant families. In addition to this, the information further indicated that such families need special attention for conservation more than the remaining families with few numbers of species. The dominance of Fabaceae in terms of species number might be because of its ubiquitous distribution since it is the third-largest family of flowering plants. The other point regarding this family is that gum-producing species belong to this family; leguminous species are members of the family, and the species of the family have different growth forms, enabling them to distribute highly in the country, which in turn enables them to be available to the different communities of the country. This and other unmentioned reasons made the family the first family in its distribution and accessibility to the local communities.

### 4.2. Growth Form Diversity

Tree, shrub, climber, and herb were the four growth forms of wild edible plants. As the review showed that trees were the primary sources of wild food [22, 25–27, 39, 41–65], shrubs took the second position as recorded by 13 articles as principal sources [21, 23, 31, 66–75]. Herbs were the least, as recorded by a few articles as main sources of wild food [64, 76–78]. Climbers were not recorded as chief sources of wild food in any article. Conversely, climbers were recorded as the second [76], the third [55, 62, 66], and the fourth [21, 23, 25–27, 42, 46, 51, 54, 57, 58, 60, 61, 67, 70, 72, 75, 77, 79, 80] source of wild food. This result showed that even if the majority of the wild food sources are trees, other edibles having other habits are also used as sources of wild food. The reason for the trees being dominant in most parts of the country might be due to their drought-resistant ability during climate change compared to other wild edible plants with other habits. It agreed with the information gathered from the reviews. Most articles recorded wild edibles as famine food. Drought is one of the causative agents of famine. Thus, this might be the reason for the trees to be the prime sources of wild food. This is because other wild edible plants with other habits, especially herbs and climbers, might not be available during drought.

### 4.3. Edible Part Diversity

**Figure 3:**
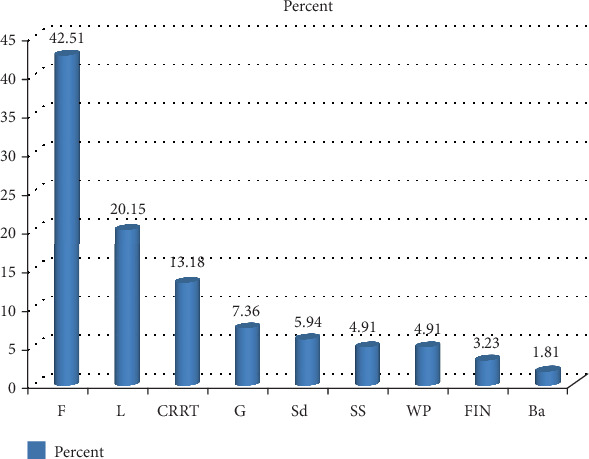
Proportion of edible parts. Note: Ba, bark; F, fruit; G, gum; L, leaf; FIN, flower, inflorescence, nectar; CRRT, corm, rhizome, root, tuber; Sd = seed; SS = stem, shoot; WP = whole part.

The results revealed that, like their species and growth form diversity, wild edible plants are varied in edible parts. The major edible parts reported are root, stem, bark, nectar, exudates such as gum, fruit, seed, leaf, and sometimes the whole part (Table 2). Fruits took the prime position to be used as sources of wild food, followed by leaves (Figure 3). This might be related to the growth forms of the edible plants, as the current review work showed that most of the species were trees, followed by shrubs, which might have the ability to set fruit.

### 4.4. Proportion of Fruits (> 75%) Compared to Other Parts by 18 Researchers

**Figure 4:**
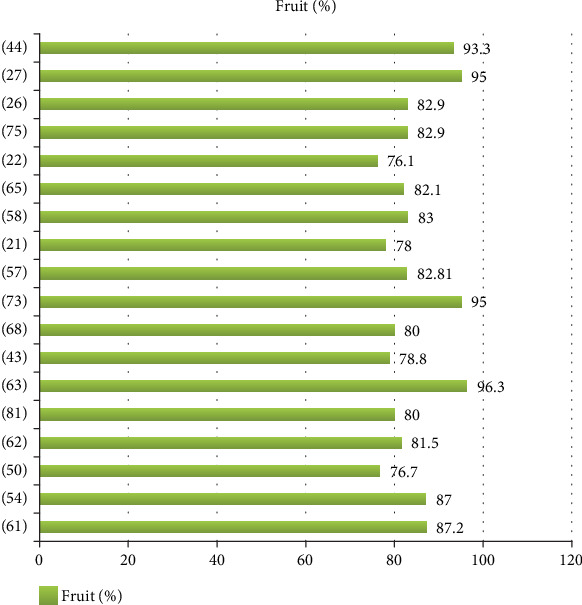
Proportion of wild edible fruits as recorded by 18 researchers.

The record of 18 researchers showed that above 75% of wild food is contributed by fruits (Figure 4), showing that fruit-producing trees and shrubs, as well as some herbaceous species, should be taken into consideration for conservation purposes.

### 4.5. Researchers With the Highest Records (> 10%)

**Figure 5:**
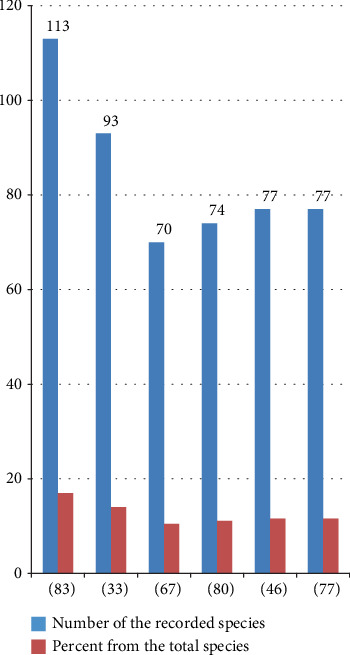
Researchers with their highest record.

The total record (665 species) was recorded by 51 researchers. Nevertheless, there was a great variation in record number, and only some of the researchers had more than a 10% share of the total record (Figure 5). This might be related to their study site variation in agroclimatic zone, vegetation cover, cultural differences of the study community in consuming wild edibles, and other additional reasons.

### 4.6. The Participating Ethnic Communities, Study Sites, Agroclimatic Zones, and Record Differences

The result showed that 26 ethnic communities were participated in the current ethnobotanical studies, i.e., (1) Afar, (2) Amhara, (3) Ari, (4) Awi Agew, (5) Benna (6) a, (7) Burji, (8) Derashe, (9) Gammo, (10) Guji, (11) Gumuz, (12) Hadiya, (13) Hammar, (14) Kara, (15) Koera, (16) Konso, (17) Kusume, (18) Kwego, (19) Maale, (20) Majang, (21) Meinit, (22) Oromo, (23) Shinasha, (24) Sidama, (25) Tigrai, and (26) Tsemay. The studies were conducted 75 districts, two at the zonal level (North Wollo and Majang zones), two in national parks (Awash and Nech Sar parks), one in a forested area (Berek Natural Forest), and two in marketplaces (Kefira market, Dire Dawa city, and Dheeraa' town). The study sites were located in eight regions of the country (Afar; Amhara; Benishangul Gumuz; Gambella; Oromia; Sidama; Southern Nations, Nationalities, and Peoples; and Tigray). In comparison, the highest records were the records of [83] at the Konso district in the Southern Nations, Nationalities, and Peoples' Region.

There was another additional record showing the rich availability of the species in the Konso district [33]. Konso is located in two agroclimatic zones (lowland and midland). The lowest record was also observed from the record of [47] at Yalo district in the Afar region, which is located in the lowland agroclimatic zone (Table 3). Additional records showed that the lowest availability is observed in the Afar region [81]. The other lowest records were observed from the record of [58] at Babile, Goro Gutu, Melka Ballo, and Meta districts and [63] at Tahtay Adiyabo, Tselemti, Tahtay Koraro, and Medebay Zana districts in the Tigray region. The record difference might be because of several reasons, including study area coverage, agroclimatic zone difference, vegetation coverage difference, cultural difference of eating edibles, and indigenous knowledge differences. For instance, the northern part of Ethiopia is highly degraded compared to the southwestern and central parts of the country. This might be the reason for the wild edible plants' record variation.

### 4.7. Top Cited Species

**Figure 6:**
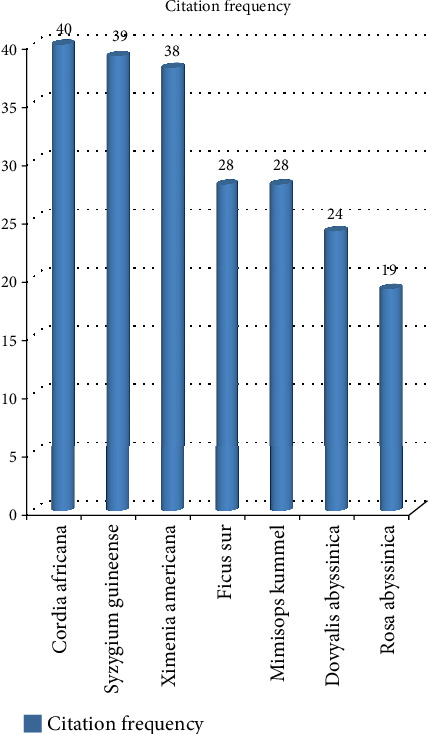
Widely distributed species list and citation.

Out of the total recorded species, some species were well known by most of the Ethiopian communities, as recorded by most of the researchers. Out of these species, *Cordia africana* was cited by 40, *Syzygium guineense* was cited by 39, *Ximenia americana* was cited by 38, *Ficus sur* and *Mimisops kummel* were cited by 28 each, *Dovyalis abyssinica* was cited by 24, and *Rosa abyssinica* was cited by 19 researchers, respectively (Figure 6). On the contrary, most species were recorded by only at least a number of researchers. For instance, species such as *Oxytenanthera abyssinica* [46] and *Sporobolus africanus* [66] were cited in a single research paper. The comparison of citation information showed the aforementioned species cited by multiple authors were the most well known as cultivated crops and highly distributed species that could be available for the diverse communities harboring several parts of the country. Moreover, it illustrates that the type of the species utilized as a source of food is different from one area to the other because of different constraints, such as differences in the culture of eating, knowledge differences (knowing whether plants are wild food or not), and the availability of that particular plant in the area.

### 4.8. Regional Record Difference (Top Regional Record)

**Figure 7:**
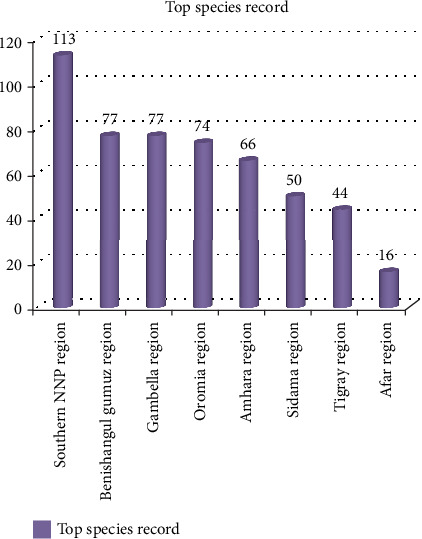
Top regional species record in Ethiopia.

The southern parts of Ethiopia harboring Konso district were the leading among several regions of Ethiopia in record number. On the other hand, a low record was observed from the Afar region in the Yalo district (Figure 7). Agroclimatically, Konso is located in two agroclimatic zones, whereas the latter district is located in a semiarid agroclimatic zone (Table 3). This might result in a record difference between the two districts. The other reason might be vegetation coverage differences and cultural differences in the consumption of wild edible plants.

### 4.9. Record Difference of Sites in the Oromia Region

**Figure 8:**
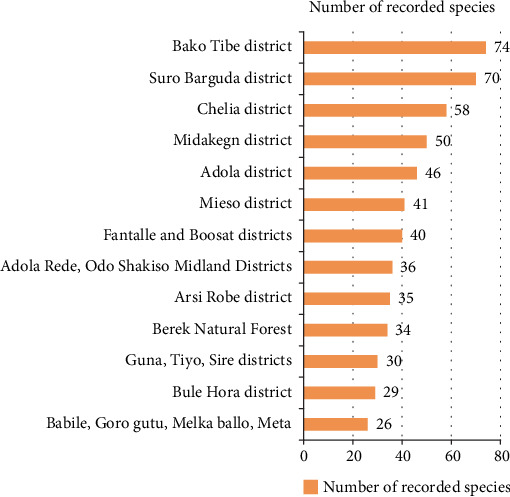
Record differences in Oromia region.

The highest record (74 species) was observed from the work of [80] from the Bako Tibe district (Figure 8). The lowest was recorded from Babile, Goro Gutu, Melka Ballo, and Meta districts by the work of [58]. As Table 3 showed, the first district is located in two agroclimatic zones (lowland and midland), whereas the latter district is located in three agroclimatic zones, which might result in a greater number of edible species. On the other hand, the result contradicts the condition, and the Bako Tibe district was the first in species record. Nonetheless, the major reason might be because of the presence of the major river, Gibe, that harbors riparian vegetation in the study area.

### 4.10. Record Difference of Sites in SNNPR

**Figure 9:**
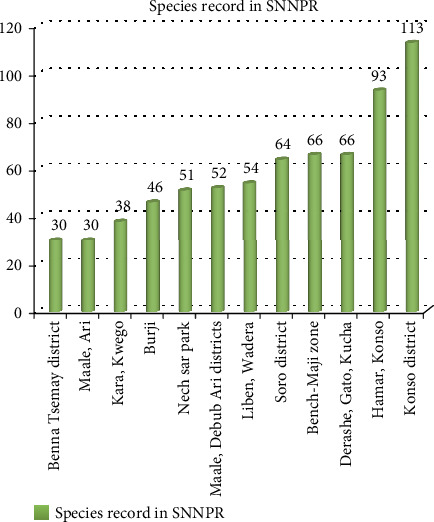
Record difference in different sites of SNNPR.

The highest record (113 species) was documented from the work of [83] from Konso districts of Southern Nations, Nationalities, and Peoples' regions (SNNPR) (Figure 9), whereas the lowest was recorded from Benna and Tsemay districts from the same region. As Table 3 showed, Benna and the location of the Tsemay district are agroclimatically semiarid, whereas the Konso district is agroclimatically located in both lowland (semiarid) and midland zones. This might be one variation for the record difference. The other reason might be that the surrounding community of Konso is still maintaining indigenous knowledge of plants, and local cultural identities are intact. Furthermore, the traditional practices of farming and agroforestry of the district harbor diverse plants, including wild plant resources [33].

### 4.11. Record Difference of the Sites in the Amhara Region

**Figure 10:**
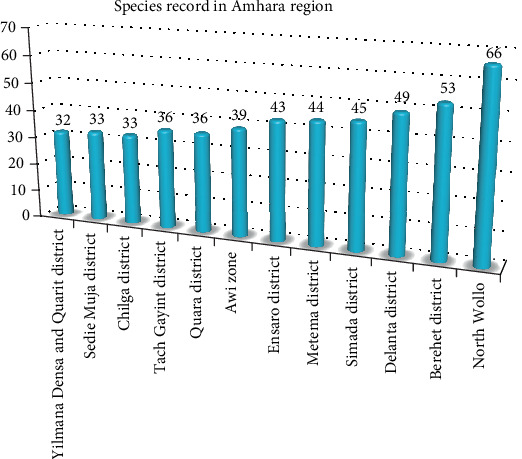
Record difference in species number in Amhara region.

The highest record of wild edible plant species (66 species) in the Amhara region was observed from the work of [51] from North Wollo (Figure 10). As [51] described, the area is characterized by a lot of beautiful irregular topography, mountain chains and peaks, flatlands, and gorges, and its altitude ranges from 980 to 4237 m above sea level. This might result in the presence of huge vegetations, which are great sources of wild edible plants. Furthermore, the site incorporates the three important agroclimatic zones (Table 3). This might result in a variation in species number.

### 4.12. Relation of Species Record With Number of Districts

It was observed that the number of species records had a linear relation with the number of districts of the study site. This might be due to the fact that as the study area increased, the number and type of species increased, and the participating population, which resulted in the culture of eating a variety of species, was also increased. However, there were some patterns that did not show such a relation.

**Figure 11:**
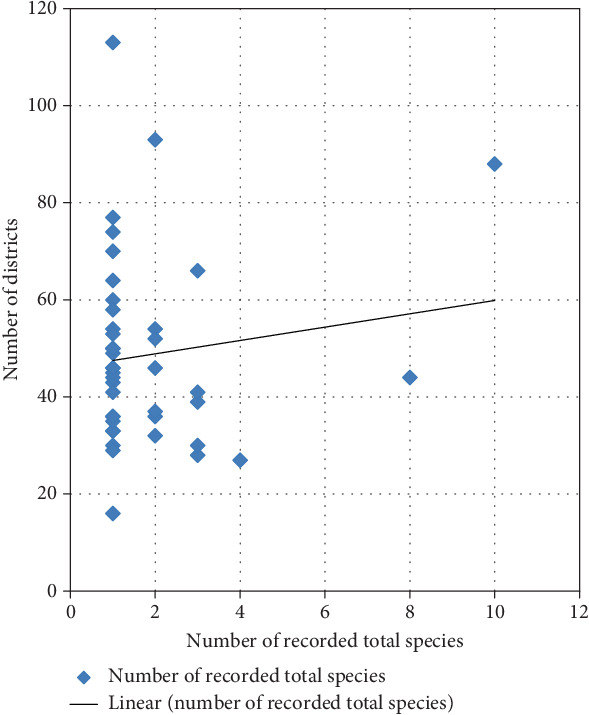
Relation of species record with the districts' number.

As some instances showed that the study site with eight districts was recorded with 60 species record while the study site with one district was recorded with more than hundred edible species (Figure 11). It further illustrates the area coverage might not matter the number of recorded species as the area or study site was inhabited by a single community with almost similar culture and knowledge in using wild edible plants.

## 5. Role of Wild Edible Plants as a Food Source

### 5.1. As Famine Food

Wild edible plants are alternative sources of food in food-insecure areas of Ethiopia, as recorded by various literature sources conducted in the country. [33] reported that the Hamer and Konso communities have experienced recurrent food shortages, which resulted in an incidence of famine. One of the means to cope with this incident is the consumption of wild edibles. In other reports by [62], 19% of the respondents responded that wild edible plants serve the community as famine food in the area. There are other additional reports that stated they were famine food [27, 86, 91]. [26] reported that wild species, *Balanites aegyptiaca*, *Cordia africana*, *Annona senegalensis*, *Rosa abyssinica*, *Cassia siamea*, and *Ziziphus mucronata* were recorded as famine foods. Also, reports further showed that they were used as the source of famine food to relieve temporal hunger [61].

### 5.2. As Sources of Proteins, Lipids, Carbohydrates, and Fibers

Studies regarding wild edible plants showed that the species were sources of proteins, carbohydrates, fats, and fibers. [31] reported that the highest crude protein content (17.47 ± 0.03 g/100 g) was found in *Amaranthus graecizans* (14.97 ± 0.03 g/100 g) whereas the highest crude fat content (14.07 ± 0.03 g/100 g) was found in the young shoots of *Rumex abyssinicus*, and the highest amount of utilizable carbohydrate (44.4 ± 0.00 g/100 g) and the estimated energy value (326.4 ± 0.00 kcal/100 g) were found in *Opuntia ficus-indica*. [32] correspondingly reported that the wild edible plants such as *Solanum nigrum*, *Dioscorea prahensilis*, and *Cleome gynandra* L. were sources of protein (4.0%–21.7%), fat (0.7%–6.1%), fiber (8.9%–22.3%), carbohydrates (38.1%–83%), and energy (275–371.1 kcal/100 g). [92] further reported that high utilizable carbohydrate and gross energy contents were recorded in *Dovyalis abyssinica*, and high protein and fat contents were observed in *Ziziphus spina christi* and *Ficus mucuso*. Moreover, [33] reported that *Coccinia grandis* was a reserve of high amounts of protein (Table 4). That might be the reason why various reports regarding wild edible plants reported them as complementary food sources.

As an example, [62] reported that 70% of the reported species were used as complementary foods for several reasons, such as pleasant tastes, though staple foods are plentiful. [62] further reported that the species were used as a temporal famine food while spending time at schools, farming places, and livestock herding, and during the scarcity of staple food staff in the area. There are other reports that further showed the complementary role of edibles as sources of food [23, 27, 58].

### 5.3. Comparison of Nutritional Content of Wild Edible Plants With Some Common Crops

Studies showed that wild edible plants are endowed with the highest nutritional contents; sometimes they have comparable nutritional contents with common crops; in some literatures, the wild edibles are recorded with even higher nutritional contents than common crops. The carbohydrate content of *Balanites aegyptiaca* was recorded as higher than that of the common crops such as *Hordeum vulgare*, *Zea mays*, *Sorghum bicolor*, *Eragrostis tef*, and *Triticum aestivum* (Table 5).

### 5.4. As Sources of Vitamin C

Reports showed that 19 species of wild edible plants were documented as sources of vitamin C. The following table (Table 6) showed that different parts of wild edible plants, such as leaves, seeds, fruits, roots, and shoots (stems), were the sources of vitamin C. The leaf (33.09 ± 0.21 mg/100 g dw (dry weight)) of *Amaranthus hybridus* was recorded to possess a higher amount of vitamin C than its seeds (2.36 ± 0.03 mg/100 g dw). The fruit (256.55 ± 9.66 mg/100 g dw) of *Carissa spinarum* was recorded to possess a higher amount of vitamin C than that of *Ficus sycomorus* L. (179.58 ± 37.64 mg/100 g dw) (Table 6). This affirmed the vitamin C content difference across species and across parts.

### 5.5. As Sources of Minerals

It was reported that wild edible plants were the sources of numerous minerals. *Rubus steudneri* were rich sources of K, Mg, Ca, Fe, and Mn [95]. High levels of Ca and Fe were recorded from *Justicia ladanoides*, Mn from *Balanites aegyptiaca*, and Zn from *Acacia ellenbeckii* were also recorded by [85]. The findings of [92] further indicated that wild edible plants such as *Ziziphus spina christi* (the fruit is a source of Fe, Zn, and Mg) and *Ficus mucosa* (high in calcium and phosphorus) were the sources of vital minerals. Likewise, the findings of [32] showed that wild edibles such as *Dioscorea prahensilis* and *Solanum nigrum* were recorded to be the sources of Na, K, Ca, Mg, Fe, and Zn (Table 7). Other studies showed that high levels of Ca and Fe were detected from the leaves of *Urtica simensis*, whereas Zn was found from the young shoots of *Rumex abyssinicus* [31].

Conversely, the other main point that should be noted is that some studies showed that wild edible plants possessing heavy metals were reported to be toxic to human health. These heavy metals, such as Cu, Cr, Ni, Cd, and Pb, were detected in the fruits of *Dovyalis abyssinica* and *Ficus sur* by [97]. Consumption of edible plants possessing these heavy metals in excess, especially Cd, is toxic to human health, resulting in renal, pulmonary, hepatic, skeletal, and reproductive effects and cancer [98]. This specifies that utilizing wild edible plants in large amounts might lead to nutritional problems and health impairment.

### 5.6. Neutraceutical Role of Wild Edible Plants

The findings of the review further showed that many wild edible plants are the sources of medicine in addition to their food source values (Table 8). The condition of edible plants as sources of medicine is known as nutraceutical plants, as defined by [7].

### 5.7. Role of Wild Edible Plants as Antioxidants

It was reported that wild edible plants were recorded to be rich sources of phytochemicals such as phenolic acids, flavonoids, alkaloids, saponins, tannins, and oxalates (Table 9), which are a potential source of natural antioxidants [110]. Sometimes, higher antioxidant activity was observed than in common crops [111]. *Amaranthus hybridus* (leaf) and *Rumex nervosus* (leaf) were recorded to have the highest flavonoid, 2,2-diphenyl-1-picrylhydrazyl (DPPH), and ferric antioxidant power (FRAP) values, scavenging 50% of free radicals under 50 *μ*g/mL [111]. [92] further reported that *Dovyalis abyssinica* was recorded with total phenolic (191.36 mg/100 g) and flavonoid (91.51 ± 3.18) contents, whereas *Ziziphus spina-christi* was recorded with high total phenolic (108.32 mg/100 g) and flavonoid (79.70 mg/100 g) contents. [95] moreover reported that the amounts of oxalate (milligrams per kilogram (kg)) in wild edible plants (ripe *Dovyalis abyssinica*, unripe *Dovyalis abyssinica*, unripe *Ficus sur*, and unripe blackberry) were 359.93, 301.01, 815.08, and 1406.15 mg/kg, respectively. The highest concentration of the heavy metals was found in blackberry, while the lowest was in unripe strawberry, as stated by [95]. In other research findings of [85], high concentrations of phenolics and tannins were recorded in *Ximenia caffra* fruits, and high concentrations of oxalates were also reported from *Amaranthus graecizans*, *Celosia argentea*, and *Portulaca quadrifida* (Table 9).

### 5.8. Role of Wild Edible Plants as Income Generation

Wild edible plants were reported to be sold in the local markets in different marketplaces in varied parts of Ethiopia. Thus, they were the source of income for the different households [25, 43, 52, 57, 62, 70, 71, 86]. According to [73], in the Mieso district's marketplace, 1 kg of fruit of *Flacourtia indica* was sold for 50 Birr (Ethiopian currency), whereas 1 kg of fruit of *Carissa spinarum* was sold for 40 Birr. Thus, they become an income source. Wild edible plants are mainly sold by females, youths, schoolchildren, and sometimes mothers [62], indicating that wild edible plants were a source of income for noncivil servants without a salary. As certain findings showed, they are the source of sustainable year-round sources of income for such community members [70].

In some parts of Ethiopia, such as the Chilga district, wild edible plants were exported to the neighboring country, Sudan, and were one of the exported goods of the country [43]. Similarly, [61] reported that *Dioscorea prahensilis* was recorded with the highest mean market price of 30.20 Ethiopian Birr (Ethiopian currency)/kg [61] further reported that the species' root tubers were available from May to early June, when there was limited rain in the region (Jawi district) and little abundance, which may cause the price to be relatively higher than that of the other marketed wild edible plants. The fruits of *Mimusops kummel* were reported and observed to be sold in a can (containing about ½ kg. A single can of its fruits was sold for 3 Birr, or, by account, 10 fruits were sold for 1 Birr [57]. The other species, the gum of *Acacia senegal*, was observed as it was sold by children and women in the villages, roadsides, and in the nearest local markets, as recorded by [91]. According to [112], *Acacia senegal* is the source of about 90% of the Arabic gum in international trade, because of its quality to that of any other *Acacias*, and it is traded for use of dyeing, ink, and medicine [112].

#### 5.8.1. Market Potential of Wild Edible Plants in Ethiopia

The result of the current findings showed that the market potential of wild edible plants in diverse portions of the country was low, as recorded by diverse research articles. According to [62], out of the reported and recorded wild edible plants, only 13% were marketable. Another study by [24] indicated that 75.7% of the recorded wild edible plants of the area were not marketed. There are other records showing the low marketability of wild edible plants [65]. The reason might be because of short production and supply [24], or it might be because of a lack of accessibility to wild edible plants in the surrounding area. In the current review work, not more than 39 species of wild edible plants were reported to be sold in the local markets of each study area (Table 10), which was much lower than the total record of the edibles. That showed that the marketability of wild edible plants in different parts of Ethiopia is still poor.

## 6. Threats

It is a communal fact that the pressures to vegetation are the threats to wild edible plants, and the driving factors are almost similar. The major threats mentioned in the review papers were agricultural land expansion resulting in the deforestation of vegetation, overgrazing, firewood collection [25, 46, 66, 77], charcoal production [75, 86], construction, tools [23, 25, 56], fuel wood collection and uncontrolled fire setting as principal threats to wild edible plants [25], and overharvesting [61]. Uncontrolled firesetting was also mentioned as the other threat by [43]. Lack of viable seed and reduced natural regeneration (for instance, poor natural regeneration of *Ficus vasta* and *Ximenia americana*), disease, insects (e.g., termites) (for example, affecting *Ziziphus spina christi* and *Balanites aegyptiaca*), poor management and protection, and settlements were further the other forms of threats [48]. In addition, drought, road construction, and urbanization were mentioned as the other major threats to wild edible plants in some parts of Ethiopia [69].

## 7. Conclusion

The review presented that Ethiopia is rich in wild edible plants. Wild edible plants are the sources of essential nutrients, minerals, medicine, and antioxidants. However, they are mostly consumed as famine food, and this might lead to the presence of low market potential for wild edible plants during a safe time. It further showed that Ethiopian communities have a great knowledge gap among themselves regarding wild edible plants, which results in consumption and management differences of the edibles in different parts of the country. The other review point is that fruits are the primary edible parts, whereas trees are the major growth forms of wild edible plants. This showed that wild edible plants are more drought resistant than other herbaceous cereal crops. In conclusion, Ethiopia has a great wealth of wild edible plants, which can supplement food shortages and feed its food-insecure community. However, the species is under major threat of extinction for multiple reasons, such as using wild edible plants only as famine food and knowledge gaps. Thus, there should be great awareness creation and introduction of wild edible plants to the local community through nurseries. Otherwise, in the future, the wild edible plant knowledge of the community will vanish because of the neglect of wild edible plants and lack of repeated practices regarding edibles. Thus, to avoid such conditions, studies regarding wild edible plants should be continued, and nurseries, especially in universities, should be established, and seedlings of wild edible plants should be distributed to the local community. Awareness creation should be conducted as well. The current findings showed that the Ethiopian flora is underutilized as a source of food. In addition, the current review paper indicated that the highest record in a single study area out of 59 study areas is 113, which is the least compared to the country's total wild edible record. Reports show that there are heavy toxic metals detected in some species of wild edible plants, which should need great care during consumption. Therefore, the knowledge regarding utilizing wild edible plants and their nutritional values should be introduced to the Ethiopian community. As an additional point, in situ and ex situ conservation and management of wild edible plants should be strengthened, and phytochemical analysis of wild edible plants should be progressed and strengthened.

## Figures and Tables

**Table 1:** Family list, species number, and percent.

**No.**	**Family list**	**Sp.N.**	**%**
1	(1) Alismataceae, (2) Anthericaceae, (3) Aquifilaceae, (4) Aponogetonaceae, (5) Balanophoraceae, (6) Balsaminaceae, (7) Berberidaceae, (8) Bignoniaceae, (9) Boletaceae, (10) Bombacaceae, (11) Canellaceae, (12) Capparaceae, (13) Chenopodiaceae, (14) Cleomaceae, (15) Costaceae, (16) Dracaenaceae, (17) Dennstaedtiaceae, (18) Erythroxylaceae, (19) Geraniaceae, (20) Hydnoraceae, (21) Hypoxidaceae, (22) Iridaceae, (23) Lobeliaceae, (24) Lythraceae, (25) Melastomaacee, (26) Menispermaceae, (27) Molluginaceae, (28) Nelumbonaceae, (29) Nyctaginaceae, (30) Ochnaceae, (31) Oliniaceae, (32) Orchidaceae, (33) Papaveraceae, (34) Phytolacaceae, (35) Pittosporaceae, (36) Plumbaginaceae, (37) Polygalaceae, (38) Resedaceae, (39) Santalaceae, (40) Simaroubaceae, (41) Thymelaeaceae, (42) Zygophyllaceae, and (43) Agaricaceae	1	0.2
2	(1) Balanitaceae, (2) Campanulaceae, (3) Clusiaceae, (4) Crassulaceae, (5) Myricaceae, (6) Moringaceae, (7) Musaceae, (8) Nymphaeaceae, (9) Oxalidaceae, (10) Podocarpaceae, (11) Portulacaceae, (12) Salvadoraceae, (13) Typhaceae, and (14) Tiliaceae	2	0.3
3	(1) Asparagaceae, (2) Asphodelaceae, (3) Cyperaceae, (4) Icacinaceae, (5) Liliaceae, (6) Loganiaceae, (7) Myrsinaceae, (8) Olacaceae, (9) Oleaceae, (10) Phyllanthaceae, and (11) Ulmaceae	3	0.45
4	(1) Apiaceae, (2) Arecaceae, (3) Asphodelaceae, (4) Cactaceae, (5) Ebenaceae, (6) Flacourtiaceae, (7) Myrtaceae, (8). Passifloraceae, (9) Polygonaceae, and (10) Urticaceae	4	0.6
5	(1) Celastraceae, (2) Combretaceae, (3) Sterculiaceae, and (4) Zingiberaceae	5	0.76
6	(1) Commelinaceae and (2) Meliaceae	6	0.9
7	(1) Convolvulaceae, (2) Sapotaceae, (3) Sapindaceae, and (4) Annonaceae	7	1
8	(1) Boraginaceae, (2) Dioscoreaceae, (3) Rosaceae, and (4) Vitaceae	9	1.4
9	(1) Araceae, (2) Rhamnaceae, and (3) Verbenaceae	11	1.7
10	(1) Apocynaceae, (2) Brassicaceae, (3) Euphorbiaceae, and (4) Rutaceae	12	1.8
11	(1) Amaranthaceae, (2) Asclepiadaceae, and (3) Burseraceae	14	2.1
12	(1) Acanthaceae	15	2.3
13	(1) Poaceae	16	2.4
14	(1) Capparidaceae	17	2.6
15	(1) Asteraceae	18	2.7
16	(1) Moraceae	22	3.3
17	(1) Lamiaceae and (2) Solanaceae	23	3.5
18	(1) Anacardiaceae and (2) Cucurbitaceae	24	3.6
19	(1) Malvaceae	28	4.2
20	(1) Rubiaceae	31	4.7
21	(1) Fabaceae	63	9.2

Abbreviations: *N*, number of families; Sp. N., number of species.

**Table 2:** Edible parts and citation.

**No.**	**Edible part**	**Citation**
1.	Fruit	[21–23, 26, 27, 41–51, 53–55, 57, 61–87]
2.	Gum	[21, 23, 26, 47, 53, 55, 57, 61, 64, 66, 67, 70, 71, 74, 78–81, 86, 87]
3.	Leaf	[21–23, 26, 27, 33, 42, 43, 45–48, 50, 51, 53, 55, 57, 58, 60–64, 66, 67, 69–72, 74, 75, 77–80, 82–84, 86, 87]
4.	Nectar	[21–23, 42, 43, 46, 47, 51, 53, 55, 57, 60–62, 64, 66, 67, 69–72, 74, 78, 79, 83, 84, 86, 87]
5.	Root	[21, 22, 26, 27, 33, 43, 46–49, 51, 55, 57, 60, 61, 64–67, 69–72, 74–76, 78–80, 82–84, 86, 87]
6.	Seed	[23, 27, 33, 42, 44–47, 50, 51, 54, 55, 57, 58, 60, 62, 64–67, 69, 71, 72, 74, 78, 80, 82–84, 86, 87
7.	Stem	[21–23, 26, 33, 42, 43, 45–47, 49, 51, 53, 55, 57, 58, 60–62, 64–67, 69, 71–75, 77–80, 82–84, 86, 87]
8.	Whole part	[22, 42, 66, 83, 86]

**Table 3:** Participated ethnic community, sites, and record differences.

**No.**	**Community**	**Study site**	**Sites**		**No. R**	**Citation**
			**Agroclimatic zone**	**Region**		
1.	Tigrai	Ambalage, Degua Temben, Endamehone, Enderta, Ganta Afeshum, Lailay Mayichewu, Raya Azebo, Tahitay Adiyabo, Tahitay Qoraro, and Tangua Abergele	Lowland, midland, and highland	TR	44	[59]
2.	Shinasha	Metekel zone (Dibatie and Bullen)	Lowland, midland, and highland	BGR	46	[60]
3.	Amhara	North Wollo	Lowland, midland, and highland	AMR	66	[51]
4.	Awi Agew	Guangua, Jawi, andAnkasha districts	Lowland and middland	AMR	39	[61]
5.	Amhara	Ensaro district	Lowland, midland, and highland	AMR	43	[72]
6.	Oromo	Adola Rede and Odo Shakiso Midland districts	Midland	OR	36	[88]
7.	Guji	Liben and Wadera districts	Lowland	SNNPR	54	[54]
8.	Benna and Tsemay	Benna Tsemay district	Semiarid (lowland)	SNNPR	30	[50]
9.	Oromo and Amhara	Kefira Market and Dire Dawa City	Not significant	OR	22	[45]
10.	Oromo	Bule Hora district	Lowland, midland, and highland	OR	29	[89]
11.	Amhara, Oromo, Agaw, and Shinasha	Dibatie district	Lowland, midland, and highland	BGR	54	[62]
12.	Afar and Oromo	Awash National Park	Semiarid (Lowland)	AFR, OR	22	[81]
13.	Sidama	Hula district	Midland and highland	SIR	50	[42]
14.	Maale and Ari	Maale and Debub Ari districts	Lowland and midland	SNNPR	52	[90]
15.	Oromo	Boosat and Fantalle	Semiarid (lowland)	OR	37	[91]
16.	Oromo	Fantalle and Boosat districts	Semiarid (lowland)	OR	40	[82]
17.	Konso	Konso district	Lowland and midland	SNNPR	113	[83]
18.	Amhara	Yilmana Densa and Quarit districts	Lowland, midland, and highland	AMR	32	[78]
19.	Gumuz	Kamashi district	Lowland	BGR	60	[69]
20.	Berta	Menge district	Lowland	BGR	60	[84]
21.	Hammar and Konso	Hammar, Konso	Lowland and midland	SNNPR	93	[33]
22.	Amhara	Metema district	Lowland	AMR	44	[79]
23.	Amhara	Berehet district	Lowland, midland, and highland	AMR	53	[86]
24.	Tigrai	Tahtay Adiyabo, Tselemti, Tahtay Koraro, and Medebay Zana	Lowland and midland	TR	27	[63]
25.	Kusume, Derashe, and Gammo	Derashe, Gato, and Kucha districts	Lowland, midland, and highland	SNNPR	66	[25]
26.	Amhara	Simada	Semiarid	AMR	45	[87]
27.	Oromo	Berek Natural Forest	Midland and highland	OR	34	[53]
28.	Tigrai	Asegde-Tsimbla, Tahitay-Koraro, and Medebay-Zana	Midland and lowland	TR	41	[70]
29.	Amhara	Chilga district	Lowland and midland	AMR	33	[43]
30.	Amhara	Sedie Muja district	Lowland, midland, and highland	AMR	33	[71]
31.	Burji	Burji district	Lowland, midland, and highland	SNNPR	46	[67]
32.	Oromo	Suro Barguda district	Highland	OR	70	[67]
33.	Amhara	Delanta district	Lowland, midland, and highland	AMR	49	[64]
34.	Oromo	Bako Tibe district	Lowland and midland	OR	74	[80]
35.	Shinasha	Bullen district	Lowland, midland, and highland	BGR	29	[76]
36.	Oromo	Mieso district	Semiarid lowland	OR	41	[73]
37.	Hadiya	Soro district	Lowland, midland, and highland	SNNPR	64	[57]
38.	Guji, Koera, and Gammo	Nech sar park (the Amaro Special Woreda and Arba Minch Zuria Woreda)	Lowland	SNNPR	51	[21]
49.	Oromo	Babile, Goro gutu, Melka ballo, and Meta	Lowland, midland, and highland	OR	26	[58]
40.	Oromo	Midakegn district	Lowland, midland, and highland	OR	50	[74]
41.	Tigrai	Tanqua Abergell, Kolla-temben, and Seharti-samre districts	Lowland, midland, and highland	TR	28	[65]
42.	Oromo	Adola district	Lowland, midland, and highland	OR	46	[22]
43.	Oromo	Arsi Robe district	Lowland and highland	OR	35	[75]
44.	Shinasha	Bullen district	Lowland (85%), midland, and highland	BGR	77	[46]
45.	Amhara, Gumuz, Berta, Hammar, and Oromo	Kobo, Dolo Mena, Raya, Homosha, Debatie, Bambasi, Mandura, Gog, Lare, and Hammar	Arid (desert), semiarid (lowland), and dry subhumid	AMR, OR, TG, GAM, BGR, and SNNPR	88	[49]
46.	Amhara	Quara district	Lowland and midland	AMR	36	[48]
47.	Oromo	Chelia district	Midland and highland	OR	58	[66]
48.	Oromo	Dheeraa' town	Not significant	OR	41	[26]
49.	Kara and Kwego	Kara and Kwego districts	Dry arid lowland (desert) and lowland	SNNPR	38	[27]
50.	Afar	Yalo district	Semiarid (lowland)	AFR	16	[47]
51.	Afar, Oromo	Awash park	Semiarid	AFR and OR	56	[23]
52.	Oromo	Mendi	Lowland and midland	OR		[41]
53.	Amhara	Tach Gayint district	Lowland, midland, and highland	AMR	36	[92]
54.	Oromo	Guna, Tiyo, and Sire districts	Lowland and highland	OR	30	[44]
55.	Majang	Majang zone	Lowland	GMA	77	[77]

Abbreviations: AFR, Afar region; AMR, Amhara region; BGR, Benishangul Gumuz region; GMA, Gambella region; No. R, number of recorded species; NR, number of recorded species; OR, Oromia region; SIR, Sidama region; SNNPR, Southern Nations, Nationalities and Peoples Region; TR, Tigray Region.

**Table 4:** Proximate composition (%) [31, 32] and [92] expressed values in mg/100 g whereas [9, 85] expressed values in g/100 g.

**Species list**	**EP**	**Moisture (wb)**	**Protein (db)**	**Fat (db)**	**Fiber (db)**	**Ash (db)**	**Carbohydrate (db)**	**Energy (kcal/100 g)**	**Citation**
1. *Adenia ellenbeckii*	L	83.6 ± 0.3	27.7 ± 0.2	NM	10.0 ± 0.6	13.8 ± 0.1	43.5	174.1	[85]
2. *Amaranthus graecizans* L.	L	72.7 ± 0.3	28.5 ± 0.2	NM	8.5 ± 0.8	22.0 ± 0.1	37.1	174.1	[85]
	L	40.8 ± 0.00^de^	14.97 ± 0.03^d^	8.40 ± 0.00^c^	9.70 ± 0.00^c^	24.7 ± 0.15^e^	1.43 ± 0.14^ab^	141.2 ± 0.23^b^	[31]
3. *Amaranthus hybridus*	Sd	9.17 ± 0.00^e^	17.63 ± 0.21	9.83 ± 0.00^e^	10.76 ± 0.01^c^	6.99 ± 0.00^a^	58.29 ± 0.21	414.80 ± 0.09^g^	[93]
	L	7.18 ± 0.07	17.63 ± 0.21b	1.88 ± 0.16	6.21 ± 0.02	15.22 ± 0.11	66.25 ± 0.21^e^	352.40 ± 1.41	[93]
4. *Amorphophallus gomboczianus*	R	84.5 ± 0.4	5.8 ± 0.1	NM	4.3 ± 0.0	6.0 ± 0.2	83.5	333.8	[85]
5. *Balanites aegyptiaca*	F	63.5 ± 1.2	28.8 ± 0.4	NM	15.5 ± 0.3	12.5 ± 0.1	40.7	162.6	[85]
6. *Celosia argentea*	L	84.1 ± 0.3	32.7 ± 0.1	NM	9.8 ± 0.1	23.9 ± 0.5	30.7	122.9	[85]
7. *Cleome gynandra* L.	F	95.6 ± 0.0^a^	20.1 ± 0.6^a^	3.3 ± 0.6^b^	22.3 ± 0.4^a^	16.4 ± 0.7^a^	41.4 ± 0.5^d^	276.0 ± 4.5^c^	[32]
8. *Coccinia grandis*	F	78.5 ± 0.7	36.3 ± 0.2	NM	10.1 ± 0.6	15.2 ± 0.1	34.9	139.6	[85]
9. *Corchorus trilocularis*	L	83.9 ± 0.0	20.4 ± 0.2	NM	11.1 ± 0.9	15.4 ± 0.8	51.7	206.8	[85]
10. *Cordia africana*	F	9.8 ± 0.00^c^	8.7 ± 0.03^c^	1.1 ± 0.00^d^	6.7 ± 0.01^b^	5.5 ± 0.03^b^	64.4 ± 0.03^a^	302.3 ± 0.12^a^	[87]
11. *Dovyalis abyssinica*	F	9.83 ± 0.01^d^	3.01 ± 0.01^d^	1.46 ± 0.08^b^	2.11 ± 0.024	0.84 ± 0.47^c^	78.27 ± 0.48^a^	342.63 ± 2.19^b^	[92]
12. *Embelia schimperi*	F	115 ± 0.03^a^	6.2 ± 0.03^c^	2.1 ± 0.02^b^	18 ± 0.03^d^	2.5 ± 0.01^b^	59.7 ± 0.27^d^	282.5 ± 2.11^d^ (kcal/100 g)	[87]
13. *Erucastrum abyssinicum*	L	8.58 ± 0.07^d^	33.63 ± 0.13^g^	1.90 ± 0.16^a^	15.53 ± 0.02^d^	18.02 ± 0.14^d^	39.50 ± 0.38	309.61 ± 0.43	[93]
14. *Erucastrum arabicum*	L	8.34 ± 0.07^cd^	30.15 ± 0.13^f^	3.80 ± 0.31^d^	21.54 ± 0.01^g^	22.75 ± 0.11^f^	30.11 ± 0.37^a^	275.17 ± 0.79^b^	[93]
	R	77.6 ± 5.8^c^	4.0 ± 0.5^d^	0.7 ± 0.1^c^	8.9 ± 1.3^c^	3.5 ± 0.1^c^	83.0 ± 0.8^a^	354.1 ± 5.4^b^	[32]
15. *Ficus mucuso* welw.ex	F	10.64 ± 0.02^b^	5.11 ± 0.24	3.31 ± 0.09^a^	0.93 ± 0.05	8.14 ± 0.11^b^	71.87 ± 0.15^c^	337.71 ± 0.51^b^	[92]
16. *Ficus vasta*	F	13.5 ± 0.002^a^	7 ± 0.03^a^	3.2 ± 0.01^c^	14 ± 0.00^b^	7.8 ± 0.002^a^	53.5 ± 0.02^d^	274.8 ± 0.06^d^	[87]
17. *Gardenia erubescens*	F	10.01 ± 0.01^c^	4.22 ± 0.38^c^	1.40 ± 0.10^c^	1.26 ± 0.05^b^	5.23 ± 0.10^c^	77.88 ± 0.14^b^	341.99 ± 0.54^a^	[92]
18. *Haplocarpha rueppelii*	L	6.95 ± 0.07	13.10 ± 0.01	2.67 ± 0.31^b^	20.71 ± 0.02^f^	12.83 ± 0.01	57.64 ± 0.24	307.02 ± 1.86	[93]
19. *Haplocarpha schimperi*	L	6.50 ± 0.07	24.04 ± 0.09^e^	3.20 ± 0.00^cd^	17.93 ± 0.02^e^	20.02 ± 0.26^e^	41.30 ± 0.13^c^	290.14 ± 0.85^c^	[93]
20. *Justicia flava*	L	80.6 ± 1.8	32.9 ± 0.5	NM	7.5 ± 0.7	25.6 ± 0.0	31.3	125.4	[85]
21. *Justicia ladanoides*	L	73.4 ± 0.5	25.4 ± 0.4	NM	12.5 ± 0.5	25.3 ± 0.3	33.9	135.5	[85]
22. *Launaea intybacea*	L	80.1 ± 1.3	24.1 ± 0.2	NM	10.7 ± 0.1	21.4 ± 0.3	40.1	160.3	[85]
23. *Leptadenia hastata*	L	76.9 ± 1.6	20.3 ± 0.8	NM	14.9 ± 0.2	13.8 ± 0.2	45.5	182.0	[85]
24. *Mimisops kummel*	F	12.8 ± 0.00^b^	3.2 ± 0.000^a^	1.86 ± 0.00^a^	19.5 ± 0.00^b^	3.1 ± 0.00^a^	60.22 ± 0.25^c^	267.7 ± 1.3^c^	[87]
25. *Pachycymbium laticoronum*	St	90.5 ± 0.8	8.1 ± 0.4	NM	15.1 ± 0.2	13.2 ± 0.2	60.5	242.1	[85]
26. *Pentarrhinum insipidum*	F	77.0 ± 0.8	32.3 ± 0.2	NM	10.9 ± 0.2	15.5 ± 0.6	38.0	151.9	[85]
27. *Portulaca quadrifida*	L	90.9 ± 0.5	19.6 ± 0.1	NM	15.9 ± 0.2	24.6 ± 0.8	36.8	147.3	[85]
28. *Rumex nervosus*	St	7.99 ± 0.13^c^	20.60 ± 0.08^c^	1.08 ± 0.00^a^	43.77 ± 0.05^h^	12.31 ± 0.11^b^	30.23 ± 0.28^a^	213.05 ± 0.79^a^	[93]
29. *Solanum nigrum*	F	88.2 ± 0.5^b^	21.7 ± 0.9^a^	4.0 ± 0.6^b^	22.3 ± 0.4^a^	14.0 ± 0.4^b^	38.1 ± 1.2^e^	275.0 ± 5.9^c^	[32]
30. *Urtica simensis*	L	9.77 ± 0.07^f^	30.55 ± 0.09^f^	3.29 ± 0.00^cd^	7.48 ± 0.02^b^	26.35 ± 0.52^g^	42.11 ± 0.38^c^	320.26 ± 1.88^e^	[93]
31. *Vigna membrancea* A.Rich	R	94.3 ± 1.5^ab^	11.8 ± 1.1^b^	4.3 ± 0.1^b^	21.1 ± 0.4^a^	12.6 ± 0.8^b^	50.3 ± 1.9^c^	286.6 ± 5.0^c^	[32]
32. *Ximenia caffra*	F	61.2 ± 0.6	21.6 ± 0.1	NM	10.4 ± 1.4	5.0 ± 0.2	39.4	157.5	[85]
33. *Ziziphus spina-christi*	F	13.10 ± 0.02^a^	5.31 ± 0.01^a^	1.65 ± 0.29^c^	0.71 ± 0.02^d^	9.23 ± 0.36^a^	70.41 ± 0.33^d^	316.05 ± 1.43^c^	[92]

*Note:* values are the mean of three independent measurements; values within a column followed by different superscripts are significantly different at *p* < 0.05 level.

Abbreviations: db, dry weight basis; EP, edible part; wb, wet weight basis.

**Table 5:** Comparison of the nutrient composition of wild edible plants with crops.

**Species list**	**Form of consumption**	**Energy (kcal/100 mg)**	**Moisture (%)**	**Protein (%)**	**Carbohy (%)**	**Fiber (%)**	**Ash (%)**	**Fat (%)**
⁣^∗^*Hordium vulgare*	Bread	158.00	52.20	4.10	36.00	2.90	1.70	1.00
⁣^∗^*Zea mays*	Bread	192.00	52.00	4.50	40.60	1.30	0.80	1.90
⁣^∗^*Sorghum bicolor*	Porridge	104.00	73.40	2.30	23.50	0.70	0.40	0.40
⁣^∗^*Eragrostis tef*	Injera	166.00	56.30	4.90	36.30	2.20	1.30	1.00
⁣^∗^*Triticum aestivum*	Bread	208.00	44.80	6.60	45.60	1.70	2.30	0.70
⁣^∗∗^*Balanites aegyptiaca*	Fruit	117.67	54.33	1.40	86.83	5.94	11.75	0.009
⁣^∗∗^*Ziziphus spina christi*	Fruit	122.38	47.59	2.13	82.04	3.78	12.09	3.722
⁣^∗∗^*Grewia flavescens*	Fruit	131.14	32.17	1.51	89.46	6.68	5.73	3.288

⁣^∗^Common crops.

⁣^∗∗^Wild edible plants [91].

**Table 6:** Vitamin C (ascorbic acid) contents of selected wild edible plants (mg/100 g dry weight basis) (NB: The values are the means of three independent composite sample analyses (on a DW basis) ± SE. At *p* < 0.05, different superscripts down the column are significantly different, and BDL means below the detection limit [94]).

**No**	**Species**	**Edible part**	**Vitamin C**	**Citation**
1.	*Amaranthus hybridus*	Grain	2.36 ± 0.03^a^	[93]
		Leaf	33.09 ± 0.21^e^	[93]
2.	*Amaranthus graecizans* L.	L eaf	180.70 ± 19.67^cd^	[94]
3.	*Carissa spinarum* L.	Fruit	256.55 ± 9.66^b^	[94]
4.	*Dioscorea alata* L.	Root	131.06 ± 25.60^e^	[94]
5.	*Dioscorea cayenensis* Lam. (yellow)	Root	259.33 ± 47.50^b^	[94]
6.	*Dioscorea prehensilis* Benth. (white)	Root	296.15 ± 33.58^ab^	[94]
7.	*Erucastrum abyssinicum*	Leaf	70.42 ± 0.14^f^	[93]
8.	*Erucastrum arabicum*	Leaf	23.31 ± 0.04^d^	[93]
9.	*Ficus sycomorus* L.	Fruit	179.58 ± 37.64^cd^	[94]
10.	*Haplocarpha rueppelii*	Leaf	BDL	[93]
11.	*Haplocarpha schimperi*	Leaf	12.77 ± 0.00^c^	[93]
12.	*Hypolepis sparsisora* (Schrad.) Kuhn.	Leaf	197.99 ± 12.78^c^	[94]
13.	*Portulaca oleracea* L.	Leaf	191.02 ± 15.83^c^	[94]
14.	*Rubus apetalus* Poir	Fruit	294.19 ± 41.90^ab^	[94]
15.	*Rumex nervosus*	Shoot	2.16 ± 0.02a	[93]
16.	*Solanum nigrum*	Fruit	126.88 ± 13.44^e^	[94]
17.	*Syzygium guineense* Wall.	Fruit	330.72 ± 27.81^a^	[94]
18.	*Tristemma mauritianum* J F Gmel.	Fruit	136.64 ± 12.77^de^	[94]
19.	*Urtica simensis*	Leaf	12.18 ± 0.02^b^	[93]

**Table 7:** Wild edible plants as sources of macro- and micronutrients.

**Wild edible plant species**	**EP**	**Calcium**	**Copper**	**Iron**	**Potassium**	**Magnesium**	**Manganese**	**Zinc**	**Sodium**	**Citation**
1. *Adenia ellenbeckii*	L	1239	0.54	16.6	NM	404	7.8	3.1	NM	[85]
2. *Amaranthus graecizans* L.	L	3029	0.65	19.3	NM	2049	7.2	2.3	NM	[85]
	L	2065 ± 195.0	NM	91.29 ± 0.75^a^	NM	NM	NM	3.81 ± 0.13^d^	NM	[94]
3. *Amaranthus hybridus*	Sd	55.01 ± 0.02^e^	NM	NM	14.40 ± 0.01^a^	70.49 ± 0.04^e^	NM	NM	37.99 ± 0.02^h^	[93]
	L	59.94 ± 0.08^g^	NM	NM	34.79 ± 0.03^d^	70.59 ± 0.20^e^	NM	NM	25.53 ± 0.03^a^	[93]
4. *Amorphophallus gomboczianus*	R	428	0.08	8.72	NM	109	1.9	1.1	NM	[85]
5. *Balanites aegyptiaca*	F	2487	0.61	13.5	NM	701	3.4	1.2	NM	[85]
	F	129.32 (± 8.96)	0.43 (± 0.06)	34.55 (± 9.29)	1541.84 (± 552.11)	NM	NM	0.47 (± 0.15)	NM	[85]
6. *Carissa spinarum* L.	F	130 ± 10.00^f^	NM	4 ± 0.53^f^	NM	NM	NM	1.33 ± 0.05^i^	NM	[94]
7. *Celosia argentea*	L	2207	1.39	19.8	NM	824	9.1	2.2	NM	[85]
8. *Cleome gynandra* L.	F	594.8 ± 32.9^a^	0.1 ± 0.0^b^	21.7 ± 2.0^b^	1487.8 ± 123.0^a^	588.1 ± 12.5^a^	NM	5.5 ± 0.04^b^	272.1 ± 0.6^a^	[32]
9. *Coccinia grandis*	F	3064	0.60	13.0	NM	433	5.6	2.5	NM	[85]
10. *Corchorus trilocularis*	L	1767.0	0.68	18.6	NM	175	8.4	2.9	NM	[85]
11. *Cordia africana*	F	94.37 (± 10.69)	0.81 (± 0.10)	15.60 (± 1.41)	1911.52 (± 85.43)	NM	NM	1.13 (± 0.27)	NM	[96]
12. *Dioscorea alata* L.	R	75 ± 5.00^f^	NM	12.83 ± 0.00^e^	NM	NM	NM	2.20 ± 0.08^g^	NM	[94]
13. *Dioscorea cayenensis* Lam. (yellow)	R	1225 ± 25.00^b^	NM	46.78 ± 0.75^b^	NM	NM	NM	3.83 ± 0.00^d^	NM	[94]
14. *Dioscorea prehensilis* Benth. (white)	R	80 ± 10.00^f^	NM	12.82 ± 2.26^e^	NM	NM	NM	2.33 ± 0.00^g^	NM	[94]
	R	3.7 ± 0.6^e^	0.1 ± 0.0^b^	3.4 ± 0.1^c^	440.6 ± 13.9^d^	68.2 ± 5.1^e^	NM	5.9 ± 0.0^a^	174.9 ± 51.5^b^	[32]
15. *Dovyalis abyssinica*	F	120.18 ± 1.18^b^	NM	2.09 ± 0.01^d^	183.36 ± 1.41^a^	5.62 ± 0.02^c^	NM	0.62 ± 0.01^d^	10.86 ± 0.17^a^	[92]
16. *Erucastrum abyssinicum*	L	49.73 ± 0.09^c^	NM	NM	43.57 ± 0.10^g^	65.31 ± 0.05^d^	NM	NM	32.86 ± 0.03^f^	[93]
17. *Erucastrum arabicum*	L	44.35 ± 0.01^a^	NM	NM	32.79 ± 0.03^c^	56.65 ± 0.24^a^	NM	NM	32.86 ± 0.03^f^	[93]
18. *Ficus mucuso* welw.ex	F	190.18 ± 0.85^a^	NM	20.96 ± 1.26^b^	165.84 ± 0.84^b^	56.55 ± 0.40^b^	NM	0.62 ± 0.01^d^	4.88 ± 0.42^d^	[92]
19. *Ficus sycomorus* L	F	321.17 ± 2.93^e^	NM	14.69 ± 0.71^de^	NM	NM	NM	4.95 ± 0.17^b^	NM	[94]
20. *Gardenia erubescens*	F	98.89 ± 0.57^c^	NM	15.04 ± 0.07^c^	107.54 ± 0.98^d^	3.44 ± 0.01^d^	NM	6.23 ± 0.06^b^	7.21 ± 0.40^b^	[92]
21. *Haplocarpha rueppelii*	L	59.05 ± 0.01^f^	NM	NM	40.77 ± 0.02^e^	62.99 ± 0.02^c^	NM	NM	28.67 ± 0.02^c^	[93]
22. *Haplocarpha schimperi*	L	49.37 ± 0.05^b^	NM	NM	54.30 ± 0.05^h^	65.14 ± 0.05^d^	NM	NM	32.46 ± 0.04^e^	[93]
23. *Hypolepis sparsisora*	L	1767.0	0.68	18.6	NM	175	8.4	2.9	NM	[94]
24. *Justicia flava*	L	3419	1.48	20.6	NM	547	8.4	2.7	NM	[85]
25. *Justicia ladanoides*	L	6177	1.17	21.2	NM	1026	7.4	3.3	NM	[85]
26. *Leptadenia hastata*	L	1699	0.59	14.2	NM	214	4.2	2.0	NM	[85]
27. *Pachycymbium laticoronum*	St	1128	0.43	13.2	NM	309	9.8	2.4	NM	[85]
28. *Pentarrhinum insipidum*	F	1100	0.41	16.3	NM	183	6.2	2.1	NM	[85]
29. *Portulaca oleracea*	L	785 ± 145.00^c^	NM	44.51 ± 8.30^b^	NM	4.33 ± 0.10^c^	NM	NM	NM	[94]
30. *Portulaca quadrifida*	L	2193	0.87	20.1	NM	1094	6.8	2.9	NM	[85]
31. *Rubus apetalus* Poir.	F	150.00 ± 20.00^c^	NM	18.48 ± 1.13^c^	NM	6.51 ± 0.08^f^	NM	NM	NM	[94]
32. *Rumex nervosus*	St	54.11 ± 0.09^d^	NM	NM	41.18 ± 0.08^f^	61.82 ± 0.08^b^	NM	NM	30.03 ± 0.06^d^	[93]
33. *Solanum nigrum*	F	241.1 ± 4.0^c^	0.38 ± 0.0^ba^	26.9 ± 13.1^ba^	1429.9 ± 14.9^a^	207.3 ± 2.6^d^	NM	3.7 ± 0.0^d^	174.9 ± 51.5^b^	[32]
34. *Syzygium guineense* Wall.	F	65 ± 5.00^f^	NM	24.90 ± 3.02^c^	NM	1.38 ± 0.25^i^	NM	NM	NM	[94]
35. *Tristemma mauritianum* J F Gmel.	F	275 ± 25.00^e^	NM	24.90 ± 2.26^c^	NM	3.56 ± 0.03^e^	NM	NM	NM	[94]
36. *Urtica simensis*	L	60.14 ± 0.05^g^	NM	NM	30.58 ± 0.07^b^	72.79 ± 0.07^f^	NM	NM	33.46 ± 0.04^g^	[93]
37. *Vigna membrancea* A. Rich	R	322.8 ± 13.6^b^	0.5 ± 0.3^a^	38.5 ± 0.2^a^	802.4 ± 83.0^c^	324.9 ± 12.9^c^	NM	3.9 ± 0.0^c^	174.9 ± 51.5^b^	[32]
38. *Ximenia caffra*	F	180	0.58	1.9	NM	110	1.1	1.3	NM	[85]
39. *Ziziphus spina-christi*	F	98.89 ± 0.57^c^	NM	15.04 ± 0.07^c^	107.54 ± 0.98^d^	3.44 ± 0.01^d^	NM	6.23 ± 0.06^b^	7.21 ± 0.40^b^	[92]
	F	170.33 (± 11.96)	0.28 (± 0.03)	11.70 (± 0.38)	1176.54 (± 471.54)	NM	NM	0.26 (± 0.08)	NM	[96]

*Note:* Values in mg/100 g.

Abbreviations: EP, edible part; F, fruit; L, leaf; NM, not mentioned; R, root; Sd, seed; St, stem.

**Table 8:** List of medicinal wild edible plants (neutraceuticals).

**No**	**Species list**	**Part used**	**Disease treated**	**Citation**
1.	*Acacia mellifera* (Vahl) Benth.	Root	Stomachache	[23]
2.	*Acacia nilotica* (L.) Willd. ex Del.	Leaf	Toothache	
		Seed	Diarrhea and cough	[99]
3.	*Acacia oerfota* (Forssk.) Schweinf.	Bark	Evil eye	[23]
4.	*Acacia senegal* (L.) Willd.	Gum	Constipation	
5.	*Acacia tortilis* (Forssk.) Hayne	Bark	Wound	
6.	*Acokanthera schimperi* (DC.) Oliv.	Leaf and stem	Syphilis and wound	[78]
7.	*Albizia gumifera* (J.F.Gmel.) C. A. Sm	Root, leaf, fruit, and stem	Helminthic (tapeworm), fungus, and trypanosomiasis	[100]
8.	*Amaranthus caudatus* L.	Root and stem	Frequent miscarriage/neonatal death	[78]
9.	*Amaranthus hybridus* L.	Leaf	Tape worm	[46]
10.	*Asparagus africanus* Lam.	Root	Babesiosis	[23]
11.	*Balanites aeygyptiaca* (L.) Del.	Fruit	Snake bite	[23]
		Leaf/Root	Abdominal pain	[46]
		Root	Malaria	
		Root	Dermal swelling	
		Root	Hypertension	
		Fruit	Diarrhea	[101]
12.	*Bidens pilosa* L.	Leaf	Tanea pedis	[46]
13.	*Brucea antidisenterica* J.F. Mill.	Leaf and fruit	Malaria, hemorrhoids, weight loss, fever, itching, and diarrhea	[102]
14.	*Cadaba farinosa* Forssk.	Root	Gonorrhea	[23]
15.	*Capparis cartilaginea* Decne.	Leaf	Intestinal parasites	
16.	*Capparis tomentosa* Lam.	Fruit	Cancer	
17.	*Carissa spinarum* (Forssk) Vahil.	Root	Tape worm	[46]
		Fruit	Constipation	
		Fruit	Gonorrhea	
		Fruit	—	[43]
		Root	Retained placenta	[103]
18.	*Clausema anisata* (Wild.)Benth.	Leaf and root	Swelling on the body	[78]
19.	*Corchorus olitorius* L.	Leaf	Diarrhea	[46]
20.	*Cordia africana* Lam.	Fruit	Diarrhea	
		Fruit	Constipation	
		Fruit	Abdominal ache	
		Fruit	—	[43]
21.	*Cordia africana* Lam.	Stem/bark	Itching	[104]
			Ascariasis, rabies, and eye disease	[99]
22.	*Cucumis ficifolius* A.Rich.	Root, fruit, and leaf	Swelling, rabies, gastrointestinal disorder, and sneezing	[78]
23.	*Cucurbita pepo* L.	Seed	Gastritis	[104]
24.	*Dovyalis abyssinica* (A.Rich) Warburg.	Leaf	Tapeworm, toothache, and sore throat	[99]
25.	*Ehretia cymosa* Thonn.	Leaf	Toothache	[23]
26.	*Ficus sur* Forssk.	Sap	Ring worm	[46]
27.	*Ficus sycomorus* L.	Sap	Hepatitis	[23]
28.	*Ficus vasta* Forssk.	Sap	Hemorrhoids	[23]
29.	*Gardenia ternifolia* Schumach. & Thonn	Root	Liver disease	[46]
			Abdominal ache (coli)	
30.	*Grewia bicolar* Juss.	Fruit	Venereal disease (syphilis)	[46]
		Root	Constipation	
31.	*Grewia villosa* Willd.	Leaf	Pasteurellosis	[23]
32.	*Hibiscus esculentus* L.	Fruit	—	[43]
33.	*Indigofera arrecta* Hochst. ex A. Rich.	Root	Snake bite	[23]
34.	*Indigofera coerulea* Roxb.	Leaf	Snake bite	[23]
35	*Justicia schimperiana* a (Hochst. ex Nees) T. Anders.	Root, leaf, fruit, and stem	Onchocerciasis and scabies	[100]
			Nausea and weight loss	[103]
		Leaf	Swelling and gastrointestinal	[78]
36.	*Lantana camara* L.	Leaf	Abdominal pain, diarrhea, nausea, and bleeding of mouth and nose	[103]
37.	*Mimusops kummel Bruce ex.DC.*	Fruit	Amoeba	[103]
38.	*Momordica foetida* Schumach.	Leaf	Bronchitis	[46]
		Leaf and stem	Gonorrhea and skin diseases	[99]
			Babies sickness	[27]
		Root and leaf	Wound infection, clotting, and snake bite	[100]
39.	*Moringa oleifera* Lam.	Leaf	Gastritis, hyperphagia, hyperdyspia, and vomiting	[103]
40.	*Ocimum spicatum* Deflers	Leaf	Eye disease	[23]
41.	*Portulaca quadrifida* L.	Leaf	Diarrhea	[46]
42.	*Prosopis juliflora* (SW.) DC	Leaf	Diarrhea	[23]
43.	*Rhamnus prinoides* L.	Leaf	Eczema	[27]
			Toothache	[105]
44.	*Ricinus communis* L.	Leaf	Impotency	[103]
		Root	Gastrointestinal disorder	[78]
45.	*Salvadora persica* L.	Root	Expel placenta	[23]
46.	*Senna occidentalis* (L.) Link	Root	Snake bite	[23]
47.	*Solanum nigrum* L.	Leaf	Abdominal pain	[46]
			Malaria	
		Fruit	Eczema	[73]
			Scabies (itching), rabies	[100]
		Fruit, leaf	Scabies (itching) and burned wound	[78]
48.	*Syzygium guineense* (Willd.) DC.	Fruit	—	[50]
		Leaf	Wound dressing, measles, and eye disease	[99]
49.	*Tamarindus indica* L.	Fruit	Diarrhea	[23]
			Abdominal pain	[46]
		Fruit	Diarrhea	[101]
			Bile and intestinal worm	[104]
50.	*Vernonia amygdalina*	Leaf	Abdominal pain	[46]
		Leaf	Constipation, flaccid paralysis, abdominal pain, and skin scraping	[103]
51.	*Ximenia americana*	Fruit	Abdominal pain	[46]
			Gastritis	
			Wound	[43]
		Leaf	Amebiasis, gonorrhea, sore throat, vermifuge, and rabies	[99]
52.	*Ziziphus abyssinica*	Root	Diarrhea	[46]
			Abdominal pain	
53.	*Ziziphus mucronata*	Leaf	Dandruff	[23]
54.	*Ziziphus spina-christi*	Leaf	Dandruff	[23]

**Table 9:** Phytochemical analysis of different wild edible plants.

**Species**	**Part**	**Phytochemicals**						**Citation**
		**Alkaloid**	**Saponin**	**Tannin**	**Phenol**	**Flavonoid**	**Terpenoid**	
1. *Albizia gumifera* (J.F.Gmel.) C. A. Sm	Bark	+++	++	++	+++	+++	++	[12]

2. *Amaranthus graecizans* L.	Leaf	++	+	+	++	+	+	[31]

3. *Balanites aegyptiaca* (L.) Del.	Fruit	−	+	N	−	−	+	[101]
		+	−	N	−	−	−	
		−	−	N	−	−	+	

4. *Brucea antidisenterica* J.F. Mill.	Fruit	+	+	+	+	+	+	[106]
	Leaf	+	+	+	+	+	+	[107]

5. *Cordia africana* Lam.	Fruit	−	+	+	−	−	N	[106]

6. *Cucurbita pepo* L.	Seed	+	+	+	−	+	+	[108]
		+	+	−	−	+	+	

7. *Ficus palmata* Forssk	Leaf	−	−	−	N	−	−	[109]
	Fruit	+	+	+	N	−	+	
		+	+	−	−	+	+	

8. *Justicia schimperiana* Hochst. ex Nees	Leaf	+	−	+	+	+	+	[107]
	Root	+	+	+	+	+	+	[12]

9. *Lantana camara* L.	Leaf	+	—−	+	+	+	+	[107]

10. *Meriandra bengalensis* (J.Koenig ex Roxb) Benth.	Leaf	−	++	N	−	−	+	[101]
		++	−	N	−	−	−	
		−	−	N	−		++	
		−	−	N	+	+	+	

11. *Momordica foetida* Schumach. et Thonn	Leaf	−	−	−	−	−	−	[100]

12. *Moringa olifera* Lam.		N	+	+	−	+	+	[103]

13. *Opuntia ficus-indica* (L.) Miller.	Fruit	+	+	+	+	+	+	[31]

14. *Ricinus communis* L.	Leaf	+	+	+	+	+	+	[107]

15. *Rumex abyssinicus* Jacq	Young shoots	+	+	+	+	+	+	[31]

16. *Solanum nigrum* L.	Fruit	++	++	+++	+	++	++	[100]

17. *Tamarindus indica* L.	Seed	−	+	−	−	−	+	[101]
		+	−	N	−	−	−	
		−	−	N	−	−	+	
		−	−	N	+	+	+	
		+	−	N	+	−	+	

18. *Urtica simensis* Steudel	Leaf	+	++	+	+	+	+	[31]

19. *Vernonia amygdalina* Del.	Leaf	N	+	+	+	−	N	[103]

*Note:* (−) absence of phytochemical, (+) mildly positive, (++) moderately positive, and (+++) highly positive (significantly visible color change).

Abbreviation: N, not detected.

**Table 10:** Major marketable wild edible plant species mentioned by different literature sources.

**No**	**Marketable wild edible plant list**	**Citation**
1.	*Acacia senegal* (L.) Willd.	[24]
2.	*Amaranthus caudatus* L.	[57]
3.	*Amaranthus hybridus* L.	[33]
4.	*Amorphophallus gomboczianus* Pichi.Serm.	[33]
5.	*Leptadenia hastata* Vatke	[33]
6.	*Balanites rotundifolius* (Tiegh.) Blatt.	[33]
7.	*Sterculia africana* (Lour.) Fiori	[33]
8.	*Balanites aegyptiaca* (L.) Delile	[23, 33, 52, 65]
9.	*Berchemia discolor* (Klotzsch) Hemsl.	[23, 24]
10.	*Borassus aethiopum* Mart.	[62]
11.	*Carissa spinarum* (Forssk) Vahil.	[57]
12.	*Cordia africana* Lam.	[65]
13.	*Dioscorea alata*	[60]
14.	*Diospyros mespiliformis*	[65, 70]
15.	*Dioscorea prahensilis*	[60, 61]
16.	*Dobera glabra* (Forssk.) Poir.	[23]
17.	*Ficus sur, Mimisops kummel*	[78]
18.	*Ficus sycomorus*	[23]
19.	*Flacourtia indica* (Burm.f.) Merr	[74]
20.	*Grewia villosa*	[23]
21.	*Grewia mollis* Juss.	[60, 62]
22.	*Hibiscus cannabinus*	[43]
23.	*Mimusops kummel* Bruce ex A.DC.	[52, 62, 71, 74, 75, 78]
24.	*Opuntia ficus-indica*	[54]
25.	*Oxytenanthera abyssinica* (A.Rich.) Munro	[61]
26.	*Portulaca quadrifida*	[60]
27.	*Rhamnus prinoides*	[78]
28.	*Rubus steudneri*	[74]
29.	*Saba comorensis* (Bojer ex A.DC.) Pichon	[60, 62]
30.	*Solanum nigrum* L.	[32]
31.	*Syzygium afromontanum* (F. White) Byng	[57, 74]
32.	*Syzygium guineense*	[52, 57, 60, 61, 70, 71, 74]
33.	*Tamarindus indica* L.	[23, 33, 43, 65]
34.	*Thymus schimperi* Ronniger	[74]
35.	*Vangueria madagascariensis* J.F. Gmel.	[33, 70]
36.	*Vitex doniana* Sweet	[60, 62]
37.	*Ximenia americana*	[24, 62, 65, 70, 74]
38.	*Ziziphus mucronata*	[26]
39.	*Ziziphus spina-christi*	[24, 26, 51, 65, 70]

## Data Availability

The data that support the findings of this study are available from the corresponding author upon reasonable request.
